# Total Meniscus Reconstruction Using a Polymeric Hybrid-Scaffold: Combined with 3D-Printed Biomimetic Framework and Micro-Particle

**DOI:** 10.3390/polym13121910

**Published:** 2021-06-08

**Authors:** Hun-Jin Jeong, Se-Won Lee, Myoung Wha Hong, Young Yul Kim, Kyoung Duck Seo, Young-Sam Cho, Seung-Jae Lee

**Affiliations:** 1 Department of Mechanical Engineering, College of Engineering, Wonkwang University, 460 Iksandae-ro, Iksan, Jeonbuk 54538, Korea; Hunjinjeong312@gmail.com; 2 Department of Orthopedic Surgery, Yeouido St. Mary’s Hospital, College of Medicine, The Catholic University of Korea, 10, 63-ro, Yeongdeungpo-gu, Seoul 07345, Korea; ssewon@naver.com; 3 Department of Orthopedic Surgery, Daejeon St. Mary’s Hospital, College of Medicine, The Catholic University of Korea, 64, Daeheung-ro, Jung-gu, Daejeon 34943, Korea; azirael99@naver.com (M.W.H.); kimtwins72@catholic.ac.kr (Y.Y.K.); 4 Department of Mechanical and Design Engineering, College of Engineering, Wonkwang University, 460 Iksandae-ro, Iksan, Jeonbuk 54538, Korea

**Keywords:** 3D printing, meniscectomy, hybrid-scaffold, polycaprolactone, tissue regeneration

## Abstract

The meniscus has poor intrinsic regenerative capability, and its injury inevitably leads to articular cartilage degeneration. Although there are commercialized off-the-shelf alternatives to achieve total meniscus regeneration, each has its own shortcomings such as individualized size matching issues and inappropriate mechanical properties. We manufactured a polycaprolactone-based patient-specific designed framework via a Computed Tomography scan images and 3D-printing technique. Then, we completed the hybrid-scaffold by combining the 3D-printed framework and mixture micro-size composite which consists of polycaprolactone and sodium chloride to create a cell-friendly microenvironment. Based on this hybrid-scaffold with an autograft cell source (fibrochondrocyte), we assessed mechanical and histological results using the rabbit total meniscectomy model. At postoperative 12-week, hybrid-scaffold achieved neo-meniscus tissue formation, and its shape was maintained without rupture or break away from the knee joint. Histological and immunohistochemical analysis results showed obvious ingrowth of the fibroblast-like cells and chondrocyte cells as well as mature lacunae that were embedded in the extracellular matrix. Hybrid-scaffolding resulted in superior shape matching as compared to original meniscus tissue. Histological analysis showed evidence of extensive neo-meniscus cell ingrowth. Additionally, the hybrid-scaffold did not induce osteoarthritis on the femoral condyle surface. The 3D-printed hybrid-scaffold may provide a promising approach that can be applied to those who received total meniscal resection, using patient-specific design and autogenous cell source.

## 1. Introduction

The meniscus is a semilunar wedge-shaped fibrocartilaginous tissue located between the articular cartilage of the femur and tibia. Meniscus tissue is composed of water, collagen (mostly type-I collagen), and glycosaminoglycans. Its major functions are protection and lubrication of the knee as well as maintenance of the overall mechanical stability by absorbing shock and stress transmission [1].

Meniscus injuries occur commonly in active populations which may develop into a small meniscus tear as well as subtotal or complete damage. Smaller meniscus tears can be repaired with suturing or commercialized cell-free (acellular) products for partial replacement of meniscus [2]. However, large meniscal tears at the inner section (white–white zone: poor blood supply) require total replacement using meniscus allograft transplantation (MAT) or collagen/biodegradable synthetic polymer-based commercial products (also known as Menaflex and Actifit^®^) [3,4,5]. Although these commercial products have shown some promising results, challenges such as weak sutures, sizing issues as well as high failure rate still remain [6,7]. Size matching of the artificial meniscus has been extensively studied to provide optimal functionality, but intraoperative size issues often occur [8]. Therefore, patient-specific shape and design are important factors for functional meniscus tissue regeneration.

Three-dimensional (3D) printing/bioprinting has emerged as a promising approach that can be tailored for use in tissue-engineered 3D constructs. Natural (e.g., collagen, gelatin, silk, and dECM bioink) and synthetic (e.g., polycaprolactone and polylactic acid) polymers have been used widely as 3D printing materials [9] and also with 3D manufacturing approaches such as electro-spinning [10]. We have examined natural and synthetic materials for use in reconstruction of the meniscus after injury or damage. Natural polymers possess a variety of advantages, including similarity to native meniscus tissue and availability of material. However, natural polymers display insufficient mechanical strength to sustain 3D shape as well as withstand external stress after implantation. For these reasons, synthetic polymeric material-based 3D-fabricated scaffolding has been utilized for total meniscus reconstruction. Polycaprolactone is especially promising in this regard because it has a low melting temperature, making it easy to control, and it also offers biocompatible/biodegradable properties.

Stem cells have been widely studied in the field of tissue engineering and regenerative medicine due to their theoretically potential advantages such as infinite differentiation and proliferation [11]. However, immune reaction issues and host cell inflammatory responses still remain, as tasks to overcome [12,13]. Hence, isolated autologous cells are attractive clinically available substitutes with biological safety. In total meniscectomy, injured meniscus tissue needs to be completely removed before inserting the artificial meniscus. In this process, autologous menisci fibrochondrocyte cell can be isolated from the removed tissues. Consequently, using this autologous cell source is the most clinically viable approach in total meniscus replacement.

In this study, we suggested and fabricated a 3D printed biomimetic hybrid-scaffold using autologous menisci fibrochondrocyte cells for total meniscus reconstruction. The hybrid-scaffold was designed in two steps: 3D bioprinting process using biocompatible synthetic polymer (polycaprolactone) to build patient-specific frameworks based on computation topological images, and then composite powder consisted of polycaprolactone (PCL) and sodium chloride (NaCl) filled into the biomimetic framework to create a microenvironment niche similar to that of the extracellular matrix. The control group used a mimetic scaffold with pore size known to be the most effective for meniscus fibrochonrocyte regeneration [14]. Meniscus fibrochondrocytes were collected from the autologous native meniscus tissue as aforementioned. The isolated fibrochondrocytes were seeded on each explant and maturing for one week of in-vitro before implantation. The in-vivo evaluations were performed using rabbit models at postoperative 6- and 12-week by histological (hematoxylin and eosin, masson trichrome, immunohistochemical) and mechanical (compressive modulus) analyses.

## 2. Methods

### 2.1. Study Design

Hybrid-scaffold was manufactured in a two-step process. First, a biomimetic PCL-framework was made using 3D printing, and then mixture particles (i.e., polycaprolactone and sodium chloride) were combined to provide a microenvironment niche similar to that of the extracellular matrix in meniscus tissue.

**Figure 1:**
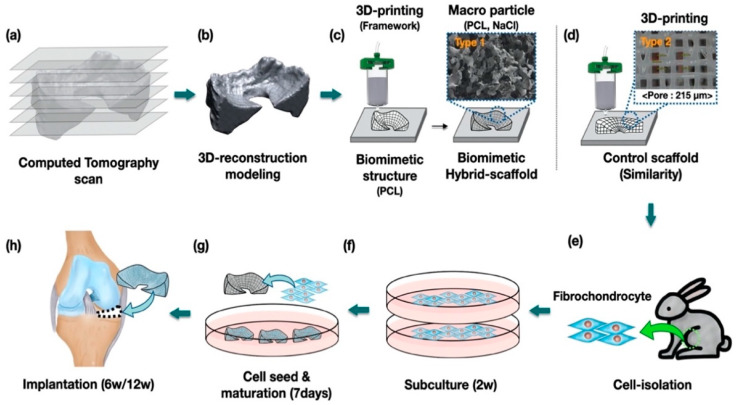
Study design and the process of preparing the hybrid-scaffold. Procedure of the (**a**–**d**) fabricated the biomimetic hybrid-, control scaffold, (**e**–**g**) cell culture and (**h**) implantation.

PCL-frameworks of hybrid-scaffold were designed with patient-specific morphological characters such as shape and size based on CT image, and then 3D-printed using reconstruction modeling procedure. (Figure 1a–c) For comparison, we prepared a control-mimetic scaffold using 3D printing. (Figure 1d) The cells were isolated from rabbit meniscus fibrochondrocyte and underwent in-vitro maturation for 7 days. (Figure 1e–g) They were implanted at the site of total medial meniscectomy in a rabbit model. (Figure 1h)

### 2.2. Fabrication of Biomimetic PCL-Frameworks of the Hybrid-Scaffold Using 3D-Printing

Mimics program (Mimics software 21.0, Materialize, Belgium) was used for reconstruction of 3D modeling of rabbit meniscus based on CT images. The reconstructed 3D modeling data was then suitably edited using a commercial Computer Aided Design (CAD) program (CATIA R13 V5, Dassault Systemes^®^, France). To generated G-code, open-source software (Slic3r, Version 1.2.9) was used to obtain the tool path for 3D printing.

To fabricate biocompatible and biodegradable frameworks, we prepared the pellet type polycaprolactone (Mn = 45,000, Sigma-Aldrich, St. Louis, MO, USA) and the lab-made precision 3D printing system. The PCL pellets were put in a stainless-steel dispenser (SS10, U-Jin Tech., Ansan, Korea) at 85 °C until fully melted. After 30 min, melted PCL was extruded at pneumatic pressure of 550 ± 10 kPa and feed rate of 4mm/sec using precision nozzles (SHN-0.15N, MUSASHI, Tokyo, Japan).

### 2.3. Fabrication of Hybrid-Scaffold with Mixture Composites

To provide a microenvironment similar to an actual extracellular matrix of the knee joint, we combined the ultrafine mixture particles (PCL and NaCl) with the fabricated PCL-frame using a Salt Leaching Using Particle (SLUP) process. For this, sodium chloride (NaCl, CAS no. 7647-14-5, Sigma-Aldrich) and polycaprolactone (Mn = 45,000, Sigma-Aldrich) were pulverized using the cryogenic grinder (Freezer/mill 6770, SPEX, Metuchen, NJ, USA) to produce ultrafine particles. The selectively used finely ground particle size of PCL and NaCl were 63~100 μm and 100~180 μm, respectively, using appropriately sized sieves. The PCL and NaCl mixed particles mixed in 1:4 (wt.%) ratio was stuffed into the PCL-frameworks. The particle size and material mixing ratio was decided based on the research results of a previous study on the SLUP scaffold [15,16]. The hybrid-scaffold combined with the mixture particles was put into the oven at 80 °C for two minutes to slightly melt the PCL particles, and then were left at room temperature to fully cool down. Lastly, the hybrid-scaffold was soaked in deionized water to leach out the NaCl particles. This fabrication process is concretely described in Supplementary Figure S1.

### 2.4. Cell Isolation of Rabbit Meniscus Chondrocyte

The meniscus fibrochondrocyte was isolated from the meniscus tissue of the New Zealand white rabbit (3 kg). After the rabbit meniscus tissue was obtained, it was washed out 3-times in PBS buffer (phosphate-buffered saline, Gibco BRL, Waltham, MA, USA) containing 1% P/S (penicillin-streptomycin, Gibco BRL) and then cut into pieces. The chopped meniscus tissue was immersed in DMEM/F12 media (Gibco BRL), which consists of the 10% FBS (Fetal bovine serum, Hyclone) and 1% P/S, 0.01% collagenase (Thermo Fisher Scientific). This solution was kept in an incubator at 37 °C for 24 h. To obtain pure meniscus chondrocyte cells, the isolated meniscus chondrocyte cells were passed through a strainer to remove remaining debris. For subculture, meniscus chondrocyte cells were incubated in DMEM/F12 cell media containing 10% FBS and 1% P/S, which was changed every three days.

### 2.5. Cell Seeding onto Scaffold

The meniscus chondrocyte cells were detached from the tissue culture plate with 1 mL 1 × trypsin-EDTA (Gibco BRL) and centrifuged at 1000 rpm for five minutes. Concentration of the meniscus chondrocyte cells was 1 × 10^6^ cells/30 μL and were seeded on the sterilized scaffolds. Cell-seeded scaffolds were incubated for seven days in the incubator before in vivo study.

### 2.6. Characterization and Morphological Analysis

The field emission scanning electron microscope (FE-SEM, S-4700, Hitachi, Tokyo, Japan) was used to observe the morphology and internal microstructure in the hybrid scaffold. The specimen was coated with platinum for 60 s for FE-SEM analysis and images were captured using beam intensity of 5.0 kV.

### 2.7. Implantation and Operative Procedures

For all surgical procedures, rabbits (New Zealand white rabbit, 3 kg) were anesthetized with Zoletil (Zoletil-Virbac, Carros, France). A 4-cm incision was made on the anterior midline of the knee from the level of the quadriceps tendon to the patellar ligament. An arthrotomy was made along the medial border of the quadriceps tendon, patella, and patellar tendon, similar to the medial parapatellar arthrotomy. The patellar tendon was dislocated laterally. To achieve full access to the medial compartment of the knee, the medial collateral ligament (MCL) was cut at its tibial insertion. After more flexion and tibial external rotation force was added, the medial meniscus was entirely exposed.

The attachments of the anterior and posterior horns of medial meniscus were transected, and the meniscus was resected at the junction between meniscus and medial knee capsule. For transplantation of cell-seeded hybrid-scaffolds, two drill holes were made using a 0.03-in. Kirschner wire in the medial aspects of the proximal tibia, ending in the anatomical attachment site of the anterior horns and midbody portion nearest from the MCL tibial attachment site. The meniscal posterior horn fixation, which is an anatomic attachment of the meniscus, was replaced with fixation of the midbody, a non-anatomic site. This was because meniscal posterior horn could not be approached without cruciate ligament transection.

The anterior horn of the meniscus was fixed with the first tibial tunnel, and the tibial re-attachment of the meniscus midbody and MCL was performed through the second tibial tunnel. Then the patellar dislocated to the lateral side was reduced, repositioned on the trochlear of the femur. The medial retinaculum was repaired, and skin suture was performed. The relevant data of the surgical procedures are described in Supplementary Figure S2.

Postoperatively, the rabbits were not set in knee casts and were closely monitored for signs of distress and pain. Intramuscular injection of gentamycin was repeatedly administered for three days. The rabbits were allowed to move freely in cages.

### 2.8. Histological Analyses

The rabbits were sacrificed at 6 and 12 weeks, and the scaffolds were immediately soaked with 10% neutral formalin and dehydrated using alcohol. These samples were embedded in paraffin to produce paraffin-blocks at 4-μm thickness. Paraffin blocks were stained with hematoxylin and eosin (H&E), IHC (collagen type I, collagen type II), and Masson’s trichrome staining (Polysciences, Warrington, PA, USA) for histochemical analyses.

### 2.9. Analysis of the Pore Size Distribution

The pore distribution in the hybrid-scaffold was measured with porosimetry (MicroActive AutoPore V 9600, Micromeritics, Norcross, GA, USA); the average value of five specimens was calculated using mercury penetration method.

### 2.10. Mechanical Property Analysis

Compression testing using a Universal Testing Machine (UTM; Model E42, MTS, Germany) was conducted for evaluation, in which Young’s modulus of in-vivo test specimens at 6- and 12-week were measured. Compression test specimens were obtained by cutting in-vivo specimens to a height of 2 mm using a surgical scalpel. The testing load cell was done at 5.0 kN, and the compressive testing speed was 0.1 mm/s. These specimens were soaked in saline before testing.

### 2.11. Animals

Standard laboratory rabbits were individually housed in wire bottom cages in a temperature- and light-controlled room. All animals were allowed to acclimate to the housing facility for 5–7 days prior to intervention and had ad libitum access to food and water. Animal care, housing, and procedures were performed in accordance with the protocol approved by the Animal Care and Use Committee of Catholic University of Korea Daejeon St. Mary’s Hospital. (CMCDJ-AP-2016-006)

### 2.12. Statistical Analysis

The data are expressed as the mean ± standard deviation. All statistical analyses were conducted using GraphPad Prism (GraphPad software; La Jolla, CA, USA). All experiments were performed in triplicate. Statistical significance was determined using two-tailed T-tests. Statistical significance was set at * *p* < 0.05, ** *p* < 0.005.

## 3. Results

### 3.1. Morphological Characteristic of the Hybrid-Scaffolds

**Figure 2:**
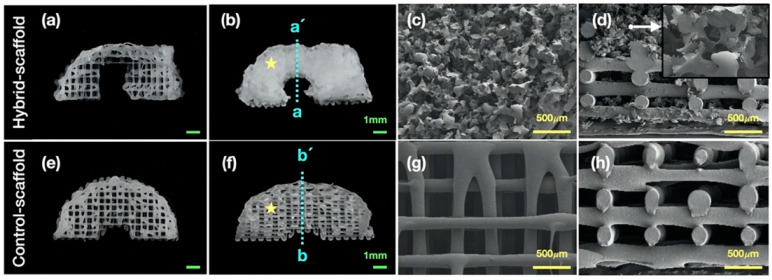
Morphology analysis, optical image of (**a**) 3D-printed PCL framework and (**b**) hybrid-scaffold, FE -SEM image of (**c**) top view (yellow star in (**b**)) and (**d**) cross-section view (a-a’). optical image of (**e**), (**f**) 3D-printed PCL framework of the control-scaffold, FE -SEM image of (**g**) top view (yellow star in (**f**)) and (**h**) cross-section view (b-b’).

The tailored-design hybrid-scaffold via the morphological data of the rabbit meniscus was successfully fabricated using the 3D printing technique. We observed the presence of irregular, superior interconnectivity-pores of 0~500 μm in the hybrid-scaffold using the FE-SEM analysis; however, the control-scaffold showed a relatively homogeneous pore structure of 215 μm (Figure 2).

### 3.2. Gross Observations

**Figure 3:**
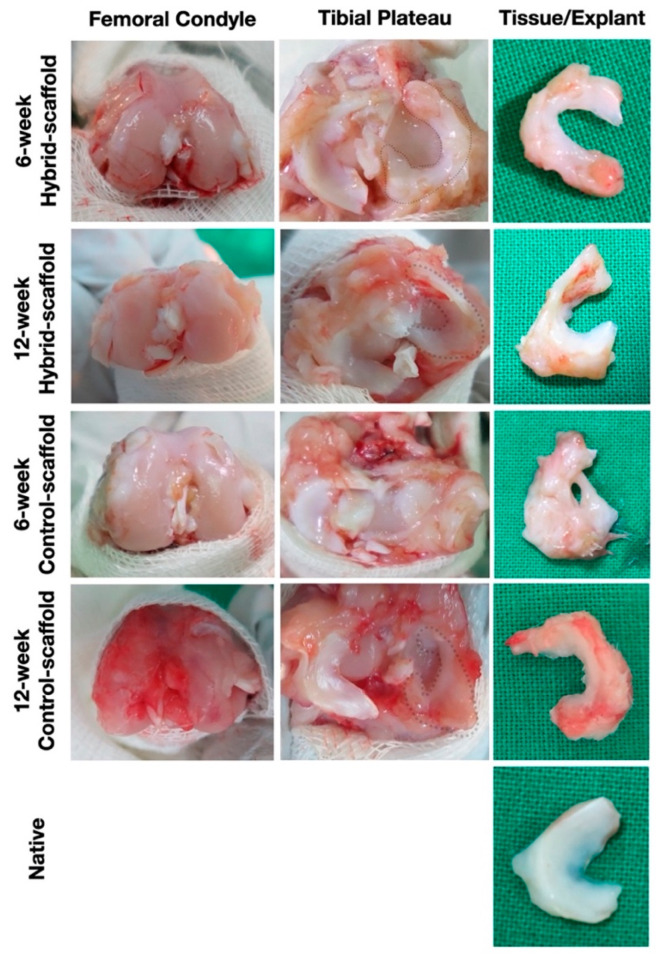
Macroscopic observations of implants at postoperative 6- and 12-week.

Macroscopically, hybrid-scaffold groups were successfully recovered at 6- and 12-weeks, which showed resemblance to the native meniscus in shape and size. Hybrid-scaffold groups showed tissue ingrowth without ruptures or fixation failures at the anterior and posterior sections. Also, there were no visible damage done to the femoral condyle surfaces. In sharp contrast, some of the control-scaffold groups were found to have distorted scaffold shape and displacement from the knee joint. At 12 weeks, the well-fixated control-scaffolds showed slight tissue ingrowth, but apparent damage to the femoral surface was observed (Figure 3).

### 3.3. Histological Analysis (Explant)

**Figure 4:**
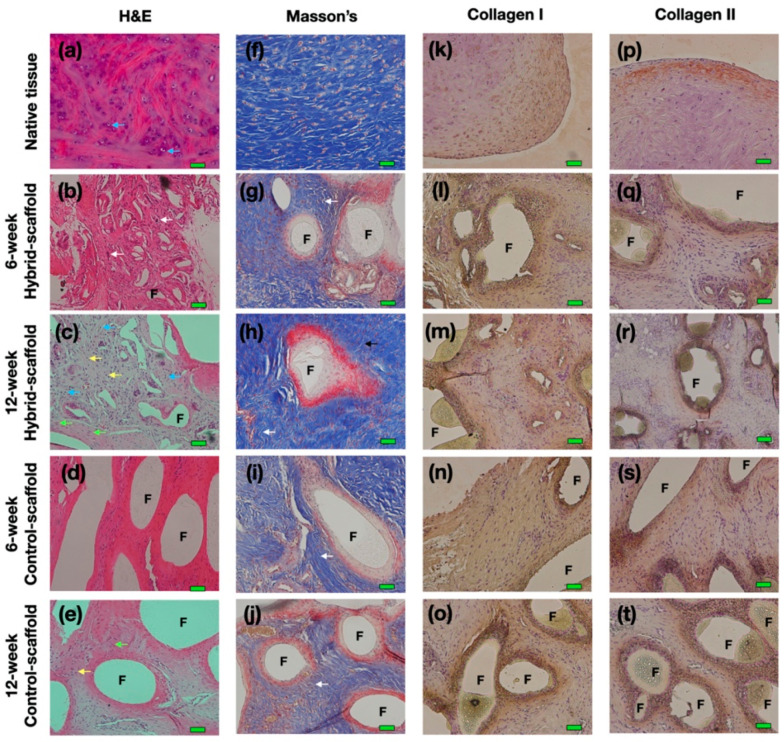
Histological analysis at 6- and 12-week explant. (**a–e**) Hematoxylin and eosin (H&E) stain, blue arrows depict mature lacunae, white arrows depict blood vessels, green arrows depict elongated fibroblast-like cell, yellow arrows depict round-shaped chondrocyte-like cells, (**f**–**j**) Masson trichrome staining, white arrows depict well-organized arrangement of collagen type I, (**k**–**t**) Immunohistochemical (IHC) staining. (magnification: ×200, scale bar: 50 μm, F: polymer fibers).

To evaluate biological properties, two-types of the scaffold were analyzed in terms of histological stain and immunohistochemical analysis. As with macroscopic observations, formation of neo-meniscus tissue was observable with cell ingrowth and incorporation. H&E staining showed elongated fibroblast-like cells (Figure 4c,d, green arrows) and abundant round-shaped chondrocyte-like cells (Figure 4c,d, yellow arrows) in both groups. Unlikely native meniscus tissues, neo-angiogenesis was also observable in hybrid-scaffold groups (Figure 4b, white arrows). The formation of mature lacunae that were embedded in the extracellular matrix were also seen. (Figure 4a,c, blue arrows). Masson’s trichrome staining showed well-organized arrangement of collagen type I with arrangement similar to that of the native tissue in both experiment groups (Figure 4f–j white arrows). Among them, the 12-week time point of the hybrid-scaffold group revealed more mature form of collagen I fiber compared to the control-groups. Immunohistochemical staining was performed to distinguish collagen type I and II. Collagen type I and II were identifiable in the neo-meniscus-like tissues of both experiment groups (Figure 4k–t).

### 3.4. Analysis of the Pore Size Distribution

**Figure 5:**
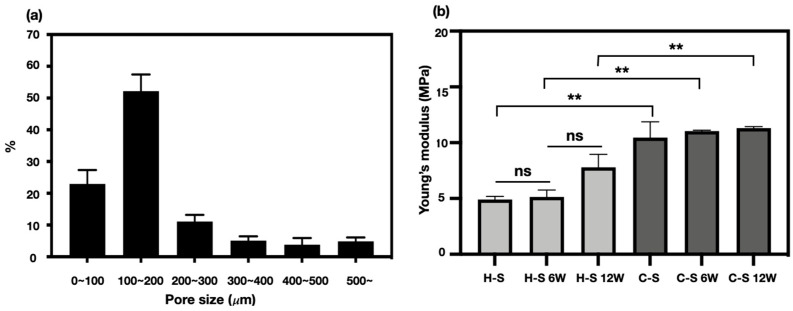
(**a**) Pore size distribution of hybrid-scaffold and (**b**) comparison of the mechanical property after implantation (** *p* < 0.005, ns: nonsignificant).

The pore size distribution test with the mercury penetration method revealed pores of various sizes in the hybrid-scaffold. Pore size of 100–180 μm occupied the largest proportion while a pore size of less than 100 μm was the second largest. Overall pore sizes varied from 200 to 500 μm (Figure 5a).

### 3.5. Mechanical Property Analysis

Using the compression test, mechanical stiffness of the hybrid- and control-scaffolds were compared after the in-vivo test at 6- and 12-weeks. Based on the stress–strain curves, Young’s modulus of the hybrid- and control-scaffolds were 4.91 ± 0.27 MPa and 10.46 ± 1.42 MPa, respectively. At 6-week postoperative, neither of the explants had significant increase in the mechanical stiffness, showing 5.14 ± 0.61 MPa and 11.04 ± 0.08 MPa, respectively. At 12-week postoperative, the hybrid-scaffold group’s mechanical stiffness increased to 7.79 ± 1.16 MPa, while that of the control-scaffold group had no significant changes (11.30 ± 0.13 MPa) (Figure 5b).

## 4. Discussion

In the field of tissue engineering, 3D printing technology has been widely utilized due to its versatile ability to fabricate desired complex free-form shape with biocompatible materials [17,18,19]. In this study, we proposed and investigated the potential of a 3D-printed, patient-specific hybrid-scaffold based on actual morphological images, utilizing autologous cells.

In the in-vivo study using rabbits that received total meniscectomy, the hybrid-scaffold was successfully recovered with preserved shape during a 12-week period. Significant cell ingrowth of neo-meniscus like cells such as fibroblasts and chondrocytes as well as matured lacunae were observed without any macroscopic inflammatory response. These results suggest that the hybrid-scaffold has significant potential as a patient-specific alternative for total meniscus reconstruction.

The major function of the meniscus tissue is protection and maintenance of the knee joint by dispersing stress from external mechanical load and shock [20,21]. The artificial meniscus structure for total meniscectomy should be designed to resist external mechanical stress applied by weight. In addition, good interconnectivity of the porous structure is essential to support efficient cell infiltration and mimic the extracellular matrix environment. To date, many commercial cell-free products using natural and synthetic polymers exist for total meniscus reconstruction [4,22].

Several researches have shown the potential of commercial products such as Actifit^®^ as a substitute for total meniscal replacement. A. Leroy et al. evaluated the functional outcome of patient who received Actifit^®^ implantation after 5 years [4]. Results showed that although cartilage status was stable at this time point, the failure rate was still high. There was also one case of implant removal due to poor function. Bahar Bilgen et. al, reported that the size matching of the artificial meniscus graft has been widely studied, but intraoperative size issues still remain an obstacle to be overcome [23]. The meniscus tissue is hard to standardize due to its individual differences. Therefore, both patient-specific size and friendly-microenvironment are critical factors that need to be considered to achieve optimal functional requirements for total meniscus regeneration.

Pores of alternative structures employed in tissue engineering are essential for excellent cell-infiltration. They facilitate nutrient distribution and promote intercellular interaction. To make a functional artificial meniscus with patient-specific design and microenvironment, we used a 3D-printing technique and salt-leaching using powder (SLUP). 3D printing is extensively used in tissue engineering because it solved the problem of size-matching with custom fabricating. Based on a previous study, SLUP method is capable of fabricating excellent porous structure similar to the extracellular matrix environment, promoting excellent cell proliferation [15,16]. The morphology analysis in hybrid-scaffold has shown superior interconnectivity. Pore size distribution test showed that pore size of 100~200 μm accounted for the highest proportion, up to 52.20 ± 5.2% of the total volume. This result occurred since the salt size of 100~180 μm has been used to create the global-pores in the hybrid-scaffold. A pore size under 100 μm and 200 to 500 μm are likely to be the result of interspacing between particle to particle, such as PCL-PCL and PCL-local pores. The control-scaffold was simply designed using a similar shape and size of the meniscus tissue. Zheng-Zheng Zhang, et al. have reported on the role of scaffold mean pore size on meniscus regeneration. In this report, a series of scaffolds with three distinct mean pore size (i.e., 215, 320, and 512 μm) were fabricated via fused deposition modeling using polycaprolactone [14]. Results showed that a mean pore size of 215 μm scaffold significantly improved both the proliferation and extracellular matrix production of mesenchymal stem cells compared to all other groups in the in-vitro study. Furthermore, the 215 μm scaffold group showed relatively better results of fibrocartilaginous tissue formation and chondroprotection in rabbit medial meniscectomy 12-week postoperative in-vivo study. Based on these results, the control scaffold group adopted the mean pore size of 215 μm.

We used meniscus fibrochondrocyte isolated from the autogenous tissue for seeding onto the scaffold. Stem cells in the field of tissue engineering are considered as a promising cell source due to their versatile proliferation ability and extensibility. Hence, several approaches have been tried using stem cells as a source for meniscus repair. In particular, the mesenchymal stem cells (MSCs), including bone marrow-derived mesenchymal stem cells (bMSCs), synovium-derived mesenchymal stem cells (SMSCs) and meniscus-derived mesenchymal stem cells (MMSCs), were widely investigated for meniscus regeneration as a promising cell source [24,25,26,27,28]. The mesenchymal stem cell is obviously the best candidate for clinical application, but it has clinical challenges including immune reactions and tissue hypertrophy [12,13,29]. In the case of patients who inevitably receive total meniscectomy, autogenous meniscal fibrochondrocyte can be easily obtained from the removed meniscus tissue during surgery. Therefore, autogenous meniscal fibrochondrocyte stands as an attractive cell source without side effects for total meniscectomy.

In terms of biomaterials, natural polymers, such as collagen, silk, and gelatin, are promising alternative materials to regenerative tissue. However, the artificial meniscus structure made using a natural polymer is insufficient to withstand external mechanical stresses. For this reason, many researchers have investigated the combination of natural and synthetic biomaterials. Merriam et al. fabricated a three-dimensional (3D) meniscus scaffold that combined collagen with hyaluronic acid and a polymer fiber for total meniscus reconstruction [30]. Similarly, Gao et al. also employed a composite scaffold that used a decellularized meniscus extracellular matrix and polycaprolactone (PCL) electrospinning fibers for mechanical reinforcement [31]. Interestingly, the use of such combined biomaterials may be considered alternative biomaterials in their own right. But tissue engineering researchers must consider approval by the United States Food and Drug Administration (FDA) for clinical trials, as the use of a complex biomaterial may complicate the approval process. In that respect, we propose a hybrid scaffold using only PCL, which is FDA approved, and autogenous cell sources. Additionally, further advantages of this hybrid scaffold are that it not only has a short manufacturing time, but is also easily controlled not requiring specialized training or expertise.

The results of histological analysis show that the hybrid-scaffold induced a more matured form of neo-meniscus tissue as compared to the control-scaffold. In particular, as shown in 12-week postoperative H&E stain, matured lacunae were identified in the hybrid-scaffold groups. Additionally, neo-angiogenesis was observed as well. These results support that intra-environment of the hybrid-scaffold fabricated with the SLUP method may provide better cell-friendly conditions due to its resemblance of the microenvironment niche in the extracellular matrix. In all experimental groups, multinuclear giant cells (MNCs) formed by foreign-body reactions were present around the PCL polymer.

Macroscopic observations revealed that the hybrid-scaffolds remain intact without extrusion from the knee joint, along with ingrowth of hyaline meniscus tissue. On the contrary, some control-scaffolds had fixation failures at the anterior and posterior sections accompanied by damage to the femoral condyle surface. The damage of the femoral condyle surface may be the result of difference in fixation method of graft substance, shape matching of the condyle surface and mechanical properties of the meniscus scaffold. Although few control-scaffold groups at 6-week postoperative had fixation failure, almost all were free of ruptures or break away. This result could support that the anchor method used in fixation of the medial aspects of the proximal tibia and midbody portion nearest from the MCL tibial attachment site, is a reliable way of maintaining grafts in place.

Ideally, the mechanical properties of implantable tissue-engineered construct should be similar to that of the target tissues or organs. The external mechanical stress threshold of the meniscus tissue is different depending on anatomic location and individual variations. Normally, the meniscus tissue disperses 50 to 100% of the imposed load on the knee joint [32,33,34]. For this reason, tissue-engineered meniscal construct for the total meniscectomy should be strong enough to withstand the load. The compressive stiffness of the hybrid-scaffold was approximately two-fold lower than the control-scaffold, measuring 4.91 ± 0.27 MPa. The compressive stiffness of the control-scaffold groups at 6- and 12-week postoperative did not significantly change. However, compressive stiffness of the hybrid-scaffold groups tended to slightly increase, although it was of no statistical significance. Consequently, it is clear that the hybrid-scaffold showed higher compressive stiffness than the native meniscus tissue (compressive modulus: 50–400 kPa) but did not induce osteoarthritis on the femoral condyle surface [35]. However, these results require further investigation on degradation rate of the biomaterials and tissue infiltration in the long term.

From the perspective of the commercial products for clinical application, hybrid-scaffold has potential advantages in its productivity. Despite the short fabrication process of one day, hybrid-scaffold can also provide tailored-design and optimal microenvironment structure. Therefore, our fabrication processes may be suitable for tissue engineering applications that require small quantity batch production.

## 5. Conclusions

It is known that the metabolism in the rabbit is three- to four-times faster than in the human. Although there are differences in the comparison of the movement mechanism of the knee joint in rabbit and human, both meniscus cartilages perform similar roles in the knee joint. Therefore, the experimental model and conditions used in this study can be encountered in human, and ultimately tissue engineering using proper scaffolds will address total meniscus defect.

In summary, we demonstrated the possibility of hybrid-scaffold as an alternative for total meniscus reconstruction. Hybrid-scaffold has shown superiority in size and shape matching as compared to the original tissue, not to mention excellent neo-meniscus cell ingrowth. Additionally, implanted hybrid-scaffold remained intact 12-weeks after total meniscectomy. The proposed hybrid-scaffold could be a promising approach for total meniscus resections.

## Data Availability

Data sharing not applicable.

## Supplementary Materials

The following are available online at https://www.mdpi.com/article/10.3390/polym13121910/s1, Figure S1. Fabrication process of the biomimetic 3D-printed hybrid scaffold; Figure S2. Surgical procedure for hybrid-scaffolds, (1) anchored to medial aspects of the proximal tibia and midbody portion nearest from the MCL tibial for (2) fixation of hybrid-scaffold;
