# Supplementary material for: Total Meniscus Reconstruction Using a Polymeric Hybrid-Scaffold: Combined with 3D-Printed Biomimetic Framework and Micro-Particle
Source: Polymers (Basel). 2021 Jun 8;13(12):1910. doi: 10.3390/polym13121910 (PMC8229034; doi:10.3390/polym13121910)
Supplement: Supplementary file 1 [file polymers-13-01910-s001.zip › polymers-1249178-supplementary (1).pdf]

Supplementary Data

Total Meniscus Reconstruction Using a Hybrid-Scaffold: Combined with 3D-printed Biomimetic Framework and Micro-Particle

Hun-Jin Jeong[1,#], Se-Won Lee[2,#], Myoung Wha Hong[3], Young Yul Kim[3], Kyoung Duck Seo[1,*], Young-Sam Cho[1,4,*] and Seung-Jae Lee[1,4,*]

[1] Department of Mechanical Engineering, College of Engineering, Wonkwang University, 460 Iksandae-ro, Iksan, Jeonbuk, 54538, Republic of Korea
[2] Department of Orthopedics, Seoul St. Mary's Hospital, Catholic University of Korea, 10, 63-ro, Yeongeungpo-gu, Seoul, 07345, Republic of Korea
[3] Department of Orthopedics, Daejeon St. Mary's Hospital, Catholic University of Korea, 64, Daeheung-ro, Jung-gu, Daejeon, 34943, Republic of Korea
[4] Department of Mechanical and Design Engineering, College of Engineering, Wonkwang University, 460 Iksandae-ro, Iksan, Jeonbuk, 54538, Republic of Korea

[#] These authors contributed equally to this work.
[*] Co-correspondence to K.D.S (kdseo85@wku.ac.kr), Y.-S. C (youngsamcho@wku.ac.kr) and S.-J. L. (sjlee411@wku.ac.kr)

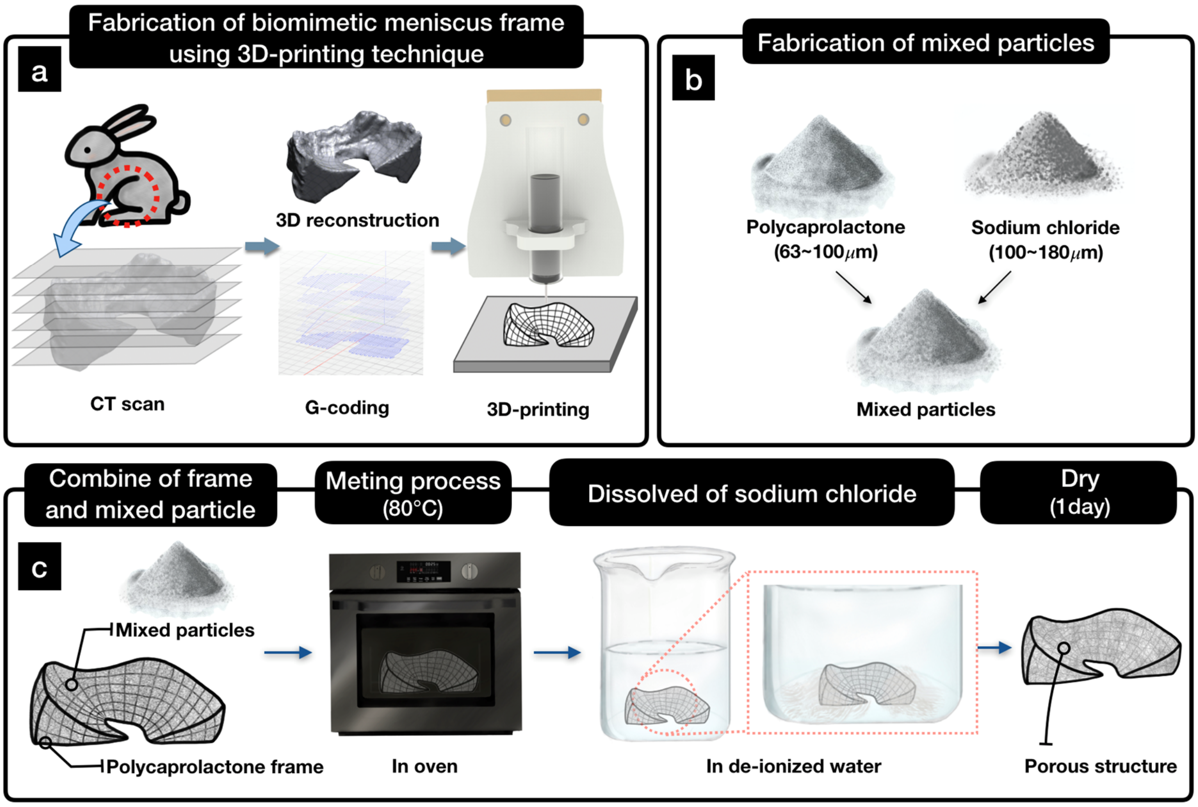

Supplemental Figure S1. Fabrication process of the biomimetic 3D-printed hybrid scaffold

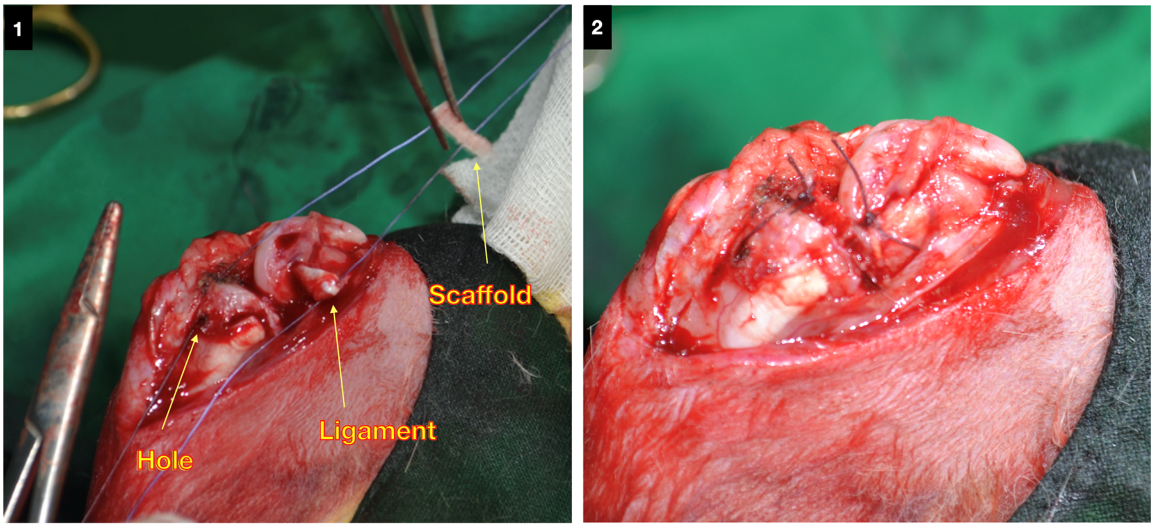

Supplemental Figure S2. Surgical procedure for hybrid-scaffolds, 1) anchored to medial aspects of the proximal tibia and midbody portion nearest from the MCL tibial for 2) fixation of hybrid-scaffold.
